# Neurofibromatosis-Associated Diffuse Lung Disease: A Case Report and Review of the Literature

**DOI:** 10.7759/cureus.8916

**Published:** 2020-06-29

**Authors:** Navdeep Dehal, Alheli Arce Gastelum, Paul G Millner

**Affiliations:** 1 Internal Medicine, Creighton University Medical Center, Omaha, USA

**Keywords:** neurofibromatosis associated diffuse lung disease, nf-dld, cystic lung disease, nf-1

## Abstract

Neurofibromatosis-1 or von Recklinghausen’s disease is an autosomal dominant disorder. Cafe au lait macules are generally the initial presenting feature of the disease, and there can be varying degrees of involvement of the skeletal, neurological, and pulmonary organ systems as the disease progresses.

The existence of neurofibromatosis-associated diffuse lung disease (NF-DLD) as a separate entity has always been questioned and, is often attributed to cigarette smoking, rather than a manifestation of NF-1.

A 59-year-old male with a history of neurofibromatosis presented with shortness of breath and ataxia for 10 days. Exam findings were pertinent for tachycardia, tachypnea, and diffuse cutaneous neurofibromas. Workup showed white blood count (WBC) of 15.9 k/ul, electrocardiogram with biatrial enlargement and right axis deviation, and a chest X-ray showed left lower lobe infiltrate concerning for pneumonia. Computed tomography (CT) scan of the chest revealed left basilar consolidation with surrounding ground-glass opacities and innumerable bilateral thin-walled cysts. The latter finding raised suspicion for NF-DLD.

The patient was evaluated by pulmonology with recommendations to continue treatment for pneumonia and follow-up with high-resolution CT of the chest and complete pulmonary function testing in 12 weeks. He was discharged in a stable condition after five days of hospitalization.

NF-DLD is a pulmonary manifestation of NF-1 with non-specific respiratory symptoms and a characteristic pattern of upper lobe cystic and basilar interstitial lung disease. It usually presents in the 4th or 5th decade, earlier in tobacco users, but a few pediatric cases have also been reported.

The presentation of NF-DLD can be variable, ranging from dyspnea, chest pain, chronic cough, hemoptysis, or an incidental finding on CT. Multiple complications, including spontaneous pneumothorax due to the rupture of subpleural blebs, pulmonary hypertension, and chronic respiratory failure, are associated with NF-DLD.

NF-DLD can be prevented by smoking cessation but, there are no known modalities for treatment; however, complications can be managed symptomatically.

This case illustrates the diagnostic challenge that NF-DLD represents to clinicians. The patient's CT from two years ago showed emphysematous changes along with scattered fibrosis and scarring, and no cystic changes were mentioned, unlike his latest CT, which showed innumerable cysts. This patient had a history of smoking, which likely put him at a higher risk for the development of cysts. However, he quit smoking 10 years prior, which suggests that his lung changes are not secondary to cigarette smoke, further confirming our suspicion for NF-DLD. Although routine screening is not implemented due to the rarity of the disease, NF-DLD should not be ruled out in patients with NF-1 presenting with pulmonary symptoms until a high-resolution computed tomography (HRCT) is obtained.

## Introduction

Neurofibromatosis-1 or von Recklinghausen’s disease is an autosomal dominant disorder. Half of the cases are inherited, while the other half develop from spontaneous mutations in the NF1 gene in the paternal chromosomes.

Genetic testing is not usually performed for the diagnosis of NF-1; it is a clinical diagnosis with the pathognomonic skin findings manifesting in the first decade. Furthermore, there is a weak genotype-phenotype correlation, and it cannot accurately predict the degree of involvement or the severity of the disease.

Cafe au lait macules are generally the initial presenting feature of the disease, followed by axillary freckling, iris hamartomas and neurofibromas. There can be varying degrees of involvement of the skeletal (pseudoarthrosis and bone dysplasia) and nervous system (peripheral neuropathy, seizures, cognitive defects, and learning disabilities). Certain tumors like neurofibromas, optic pathway gliomas, malignant peripheral nerve sheath tumors (MPNSTs), rhabdosarcoma, gastrointestinal stromal tumor (GIST), and pheochromocytoma are commonly associated with the NF-1. The thorax and lungs can also be involved in NF-1 and can present as cutaneous neurofibromas, kyphoscoliosis, pulmonary hypertension, pulmonary artery stenosis, neurofibromatosis diffuse lung disease (NF-DLD) and rarely adenocarcinoma of the lung.

The existence of NF-DLD as a separate entity has been questioned ever since it was first reported and, more often than not, is attributed to cigarette smoking, rather than a manifestation of NF-1. In this manuscript, we present the case of a patient with NF-1 who was diagnosed with NF-DLD.

## Case presentation

A 59-year-old male with a 30-year history of neurofibromatosis presented with shortness of breath and ataxia for the last 10 days. Other past medical history includes tobacco abuse with 30 pack-year history, quitting 11 years prior to presentation. On arrival to the emergency department (ED), he was tachycardic with a heart rate of 101 beats per minute and tachypneic with a respiratory rate of 24 breaths per minute. The rest of his vital signs were within normal limits. His physical exam revealed no acute distress; he was awake, alert, and oriented to person, place, and time. Skin exam revealed multiple soft cutaneous neurofibromas and cafe au lait spots. Lung auscultation revealed good air entry bilaterally. Cardiac examination revealed a regular rate and rhythm, with no murmurs or gallops appreciated. Laboratory test illustrated a white blood count of 15.9 k/ul, hemoglobin of 12.2 gm/dl, glomerular filtration rate (GFR) >90 mL/min/1.73 m^2^, glucose of 66 mg/dl, blood urea nitrogen (BUN) of 12 mg/dl, creatinine of 0.68 mg/dl, sodium of 136 mmol/dl, potassium of 3.5 mmol/L, chloride of 104 mmol/L, CO2 of 26 mmol/L, calcium of 8.6 mg/dl, aspartate aminotransferase (AST) of 19 u/l, alanine aminotransferase (ALT) of 26 u/l, alkaline phosphatase of 275 u/l. An electrocardiogram showed biatrial enlargement and rightward axis deviation, compatible with pulmonary disease pattern.

**Figure 1:**
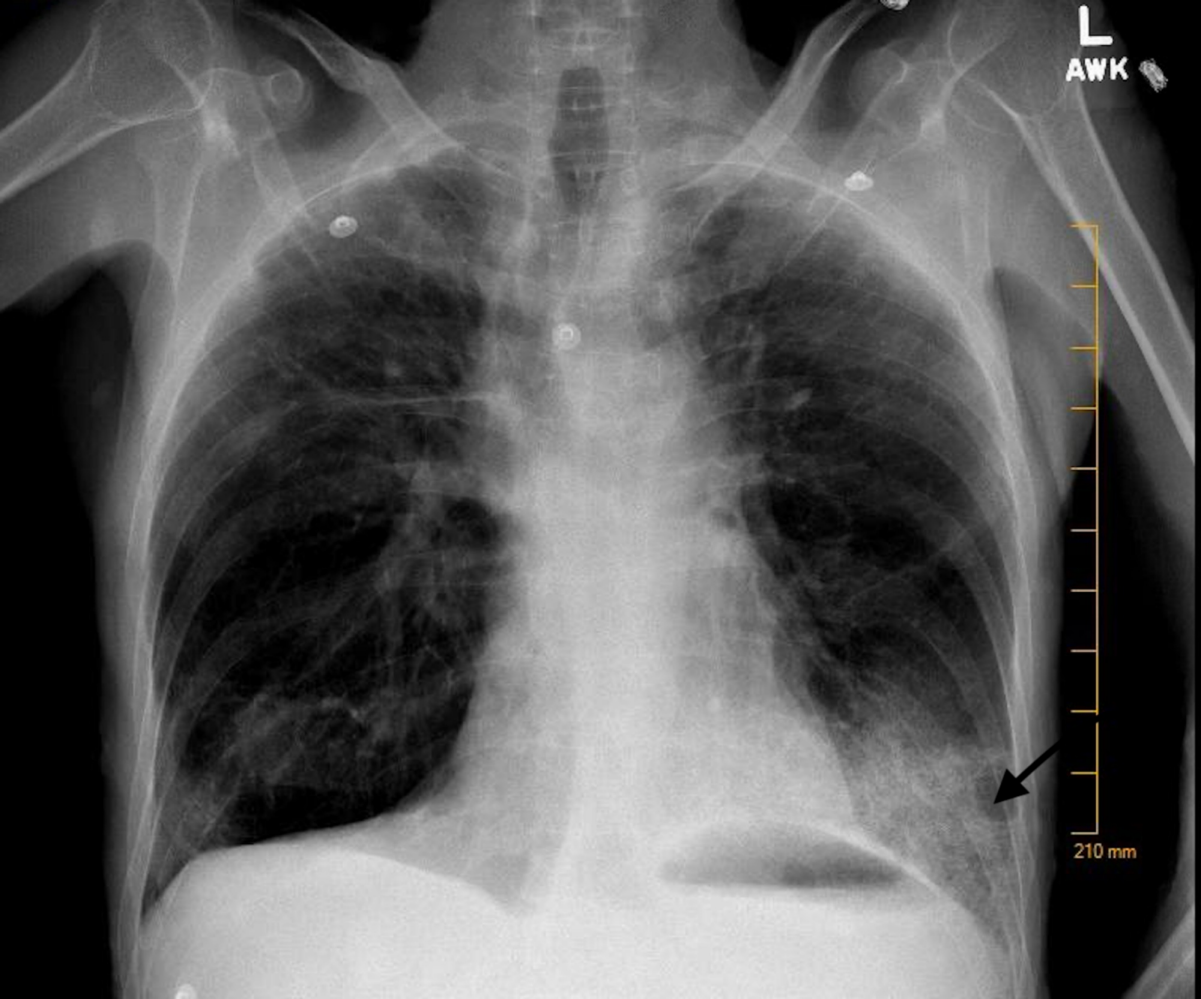
Chest X-ray showing left lower infiltrate.

**Figure 2:**
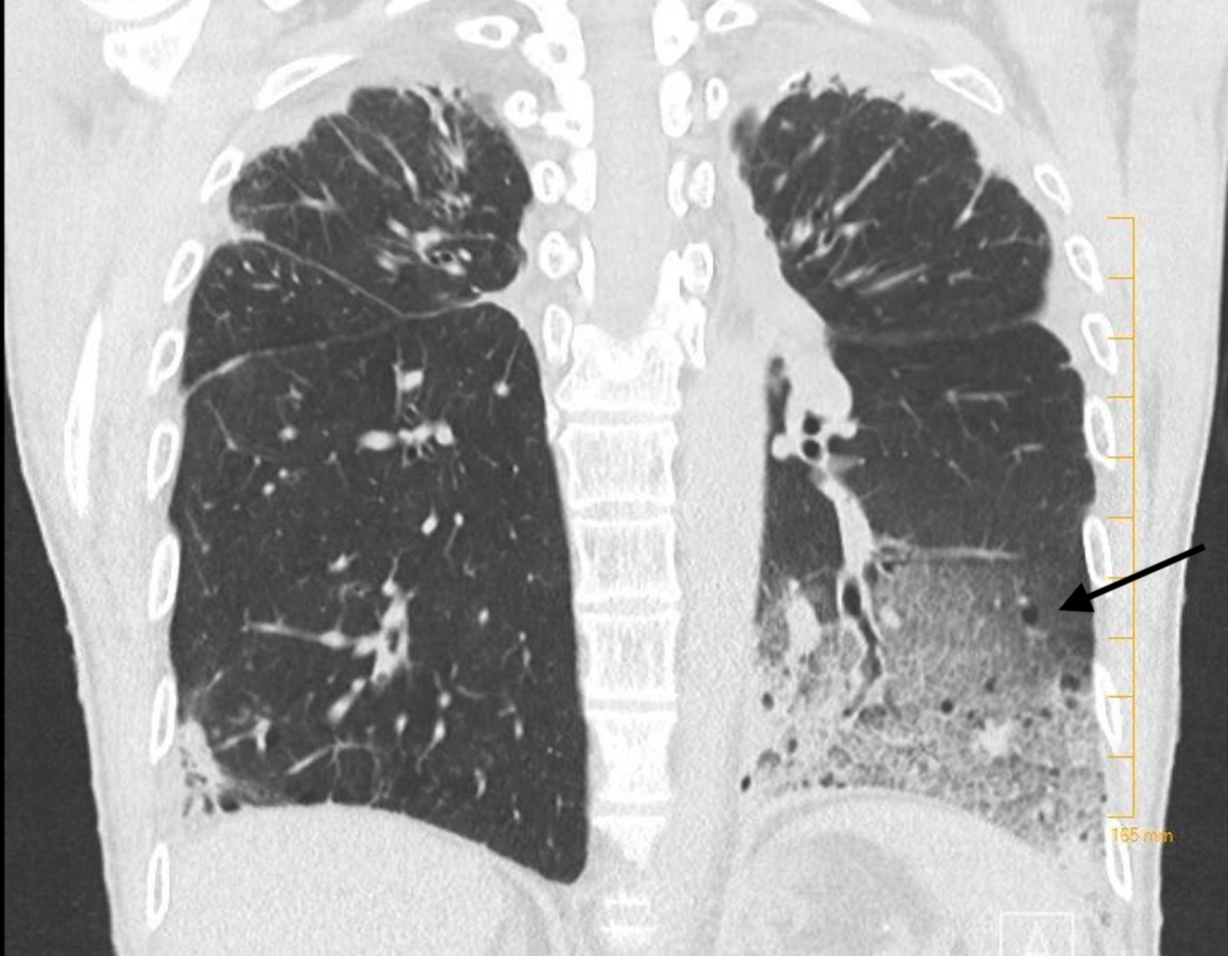
CT chest showing multiple cysts and left basilar consolidation with ground glass opacities.

**Figure 3:**
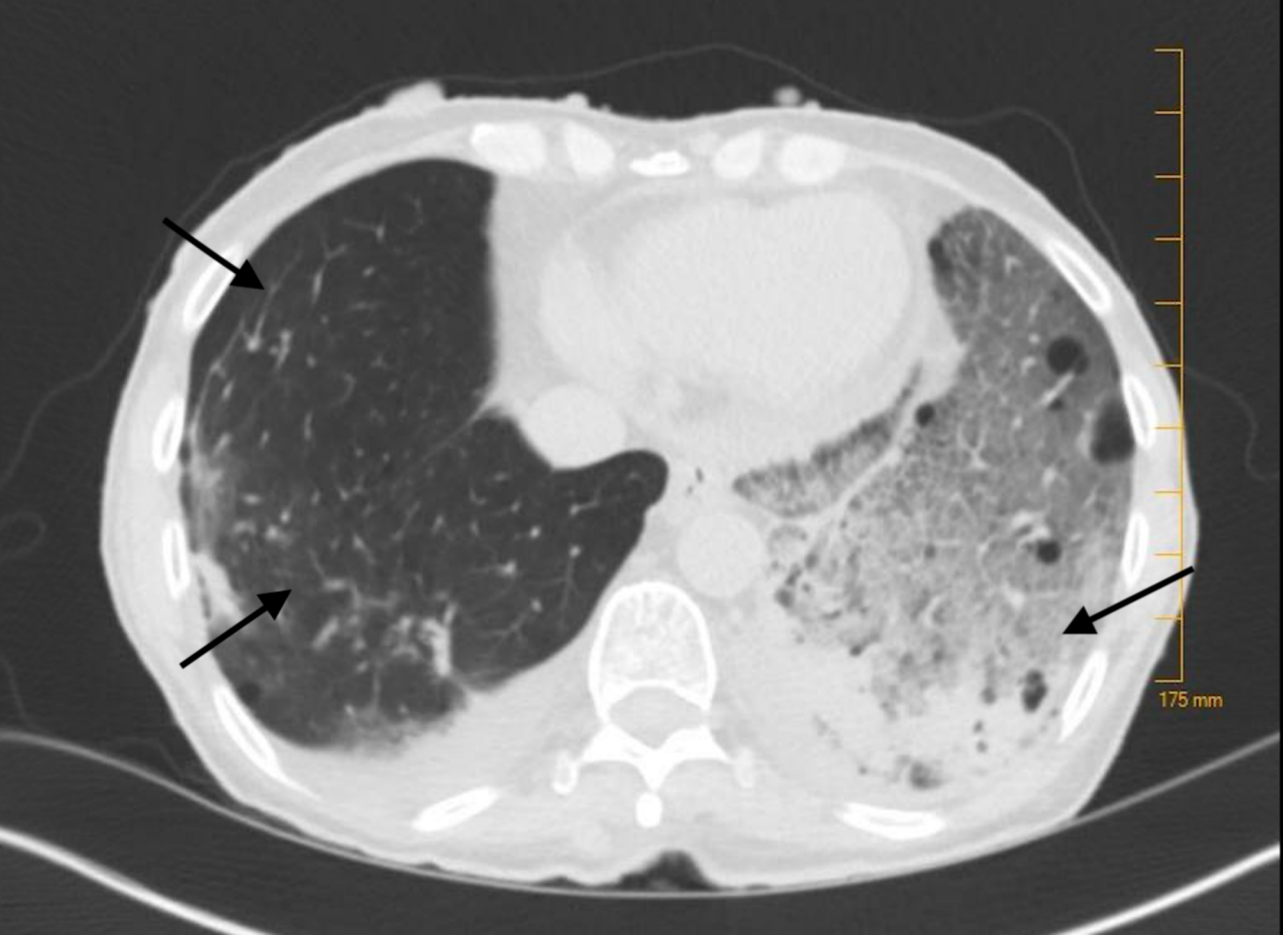
CT chest showing multiple cysts and left basilar consolidation with ground glass opacities.

A chest radiograph revealed a left lower lobe infiltrate concerning for pneumonia (Figure 1), and a computed tomography (CT) scan of the chest was recommended. CT of the chest was positive for left basilar consolidation with surrounding ground-glass opacities and innumerable bilateral thin-walled cysts (Figures 2, 3). The latter finding raised suspicion for neurofibromatosis-associated diffuse lung disease (NF-DLD). On chart review of previous hospital admissions, a CT of the chest from two years ago showed emphysema with scattered fibrosis and scarring, but no cysts were reported. Intravenous fluid resuscitation and antibiotics were initiated. The patient was admitted with sepsis secondary to community-acquired pneumonia.

After consultation with pulmonology, recommendations were to continue treatment for pneumonia and follow up with high-resolution CT of the chest and complete pulmonary function testing in 12 weeks.

The patient’s ataxia was investigated by neurology; CT and magnetic resonance imaging (MRI) of the brain were unremarkable. The ataxia was thought to be secondary to sepsis. His balance improved after the infection was treated. Follow-up with the movement disorder clinic was recommended. After seven days of hospitalization, the patient was discharged in a stable condition with close follow-up with pulmonary specialists.

## Discussion

NF-DLD is a pulmonary manifestation of NF-1 with non-specific respiratory symptoms and a characteristic pattern of upper lobe cystic and basilar interstitial lung disease. It usually presents in the fourth or fifth decade, earlier in tobacco users, but a few pediatric cases have also been reported.

A study done by Riccardi in 1981, observed 200 patients with NF-1, and none were found to have pulmonary parenchymal disease. However, no CT scans were performed on the enrolled patients, thereby possibly missing the diagnosis [1]. Zamora et al. performed a retrospective case series, where they reviewed 64 patients with NF-1. Eight people had high-resolution computed tomography (HRCT) scans, out of which 25% showed emphysema and cysts, 37% showed ground-glass opacities, and 50% showed reticular abnormalities and bullae. Honeycombing, as is typically seen in advanced fibrotic interstitial lung disease (ILD), is notably absent [2].

Pathophysiology

The NF-1 is a tumor suppressor gene, which encodes for neurofibromin, a protein that downregulates the Ras activity. Mutations in the NF-1 gene can lead to decreased or a malfunctioning neurofibromin production, which is unable to suppress Ras activity and thereby increasing cell growth and proliferation.

The exact pathophysiology behind NF-DLD is unknown, but it can be hypothesized from previous studies that it is due to increased collagen deposition. A study by Patchefsky et al. found areas of healthy lung alternating with areas of fibrosis, septal thickening, and increased cellularity. Amyloid deposition was seen in some cases as well [3]. The cysts form from increased lymphocytic infiltrates around the alveolar septa obstructing and dilating the bronchioles, leading to cyst formation [4].

Clinical presentation

The presentation of NF-DLD can be variable and is often non-specific. Symptoms can range from dyspnea, chest pain, chronic cough, hemoptysis to spontaneous pneumothorax, or it can be an incidental finding on CT.

Pulmonary function tests can vary from obstructive, restrictive to mixed or normal patterns, but diffusing capacity of the lung for carbon monoxide (DLCO) is always reduced [2].

Smoking and NF-DLD

It has been long disputed that the cause of diffuse lung disease in NF-1 is secondary to smoking. A study performed by Ueda et al., where they looked at chest CT findings of 88 patients with NF-1, did not show a significant difference in the occurrence of cysts between smokers and non-smokers [5].

Another study by Oikonomou et al. compared chest CTs of six never smokers with NF-1. All six subjects were found to have thin-walled cysts ranging from three to >100 in number. They also concluded that although smoking is not the cause of diffuse lung disease in NF, it can increase the severity of the disease and manifest at a much younger age [6].

NF-DLD has also been reported in the pediatric population in a four- and a 16-year-old non-smoker [7]. The first was an incidental finding of numerous cysts in the apical lungs while undergoing a CT spine for evaluation of scoliosis. The 16-year-old patient presented with pneumothorax, and his CT showed bilateral apical bullae. He was treated with a thoracostomy tube insertion, followed by video-assisted thoracoscopic surgery (VATS) for apical bullae resection and partial pleurectomy [8].

Histopathological findings of lung biopsies from patients with NF-DLD show elevated intra-alveolar eosinophils and pneumocytes instead of macrophages, as expected in respiratory bronchiolitis and desquamating interstitial pneumonia associated with smoking [9].

HRCT findings in NF-DLD show thick and well-defined borders around the cysts and bullae, unlike emphysema secondary to cigarette smoking, where the borders are ill-defined. The cysts observed in NF-DLD are a result of distal acinar emphysema as compared to centriacinar emphysema seen in smokers [10].

A case report from 2012 evaluated a 34-year-old male with NF-1, HIV, and a 16-pack-year history of smoking presenting with dyspnea and chest pain. On comparing CT scans done seven years apart, it was noted that there was a rapid progression of diffuse lung disease with the typical pattern of cystic disease in the upper lobes and bibasilar ground-glass appearance [11]. The conclusion that smokers with NF-1 develop NF-DLD at an earlier age as compared to non-smokers was drawn given the typical CT scan appearance. Furthermore, cessation of smoking can lead to disease stabilization, as seen by Miyamoto [12].

Complications

Multiple complications, including spontaneous pneumothorax due to the rupture of subpleural blebs, pulmonary hypertension, and chronic respiratory failure, are associated with NF-DLD [13, 14].

NF-DLD can progress to pulmonary hypertension (PH-NF1) and is classified as group 5 PH (unclear/multifactorial etiology). Factors thought to play a role in PH-NF1 are underlying parenchymal lung disease, vascular remodeling of small blood vessels, and restrictive disease caused by skeletal deformities [4].

Lung parenchymal changes, including cysts/bullae and ground glass/reticular opacities, are seen in about two-thirds of cases with PH-NF1. The remaining one-third of cases are attributed to underlying vasculopathy seen with NF-1 [1].

There are studies linking adenocarcinoma of the lung to NF-DLD. Still, there is a paucity of cases in the literature, and further studies are required to establish the relationship between the two [15].

Management

Presently, there are no known modalities for the prevention or treatment of NF-DLD; however, complications can be managed symptomatically. Routine screening for NF-DLD is not implemented due to the rarity of the disease, but all patients with NF-1 who present with respiratory symptoms should undergo HRCT.

Furthermore, patients should be counseled about smoking cessation as it could slow the progression of the disease [11,12].

## Conclusions

This case illustrates the diagnostic challenge that NF-DLD represents to clinicians. The patient’s CT from two years ago showed emphysematous changes along with scattered fibrosis and scarring, no cystic changes were mentioned, unlike his latest CT, which showed innumerable cysts. Our patient had a history of tobacco abuse, which likely put him at a higher risk for the development of cysts. However, he quit smoking 10 years prior, suggesting that his lung changes are not secondary to cigarette smoke, further confirming our suspicion for NF-DLD.

Hence, NF-DLD should not be ruled out in patients with NF-1 presenting with pulmonary symptoms until an HRCT is obtained.
